# The Relationship Between Satisfaction With Life and Depression Symptoms by Gender

**DOI:** 10.3389/fpsyt.2019.00419

**Published:** 2019-06-14

**Authors:** Antonella Gigantesco, Corrado Fagnani, Virgilia Toccaceli, Maria Antonietta Stazi, Fabio Lucidi, Cristiano Violani, Angelo Picardi

**Affiliations:** ^1^Centre of Behavioural Sciences and Mental Health, Italian National Institute of Health, Rome, Italy; ^2^Department of Psychology of Development and Socialization Processes, Sapienza University of Rome, Rome, Italy; ^3^Department of Psychology, Sapienza University of Rome, Rome, Italy

**Keywords:** depressive symptoms, subjective well-being, satisfaction with life, gender, mental health policy

## Abstract

Depression is a worldwide public health concern. The World Health Organization (WHO) has recently recommended the implementation of programs for strengthening subjective well-being (SWB) to reduce mental disorders, including depression. Also, in 2013, European member-states agreed on a single measure of SWB, i.e., life satisfaction, for monitoring the progress of SWB in the WHO health policy framework, “Health 2020.” Life satisfaction is strongly associated with depression; therefore, its use as health indicator could be suitable to identify individuals at risk for depression. Critical to this use of life satisfaction to target also depression is knowledge on the nature of the association between the two throughout the lifespan and by gender. This study aims at contributing to the knowledge about this association in a sample of 51 individuals screened for major depressive disorder (MDD) and dysthymic disorder (Dys). All individuals were administered the Primary Care Screener for Affective Disorders and the Satisfaction With Life Scale (SWLS). Among individuals negative for MDD or Dys, women displayed similar satisfaction compared with men, whereas among individuals positive for MDD or Dys, women showed greater satisfaction compared with men, whose score denoted life dissatisfaction. Consistently, the regression model for SWLS revealed a significant main effect of positivity for MDD or Dys on life satisfaction as well as a significant interaction between positivity for MDD or Dys and gender. The results of this study do not support the notion that satisfaction with life and depressive symptoms could belong to highly related dimensions, at least among female individuals.

## Introduction

Mental and substance use disorders, led by depressive disorders, are a major cause of non-fatal burden of disease worldwide (1). They accounted for an estimated 7.4% of disability adjusted life years (DALYs) and 22.9% of years lived with disability (YLDs) in 2010 (2). According to the World Health Organization (3), major depressive disorder (MDD) affects approximately 350 million people worldwide each year and represented the fifth leading cause of worldwide disease burden, accounting for 12% of all YLDs in 2016 and contributing 34.1 million of total YLDs (1). MDD is expected to become the leading cause of the disease burden worldwide by 2030 (3). This disorder is associated with higher medical costs, increased physical symptoms burden, greater impairment in social functioning, poorer self-care, and adherence to medical treatment in patients with comorbid physical illness (4). In the United States, the cost of MDD was estimated for the year 2000 at $83.1 billion (5).

Despite this, only a proportion of those who suffer from MDD are reached and diagnosed by health care systems. In Europe, the European Study on the Epidemiology of Mental Disorders (ESEMeD) project (6) found that among individuals who had experienced MDD, dysthymia, anxiety, or alcohol disorder within the previous 12 months, only about one in four reported using formal health services, and that among the six participating European countries, Italy had the lowest usage of health services.

In the light of this, a clear public health goal is to intercept individuals at high risk for developing MDD in order to deliver them effective preventive interventions. Improving the ability and accuracy of screening procedures may lead to better identification and treatment of depression. Such procedures require the involvement of mental health professionals, specialized medical settings, and the integration of primary care (PC) and mental health services for implementation, evaluation, and sustainability (7).

By a broader public health point of view, a comprehensive approach must include interventions targeting community populations, not only individuals in medical settings. Combining public health initiatives with medical discipline interventions has been found to be functional to implement a multilevel approach for mental disorder prevention in the general population (8, 9). Whereas the effects of medical disciplines and interventions are assessed among individuals who attend medical settings, the evaluation of the impact of community public health interventions might be accomplished by linking public health initiatives to on-going national tracking surveys, like the Behavioural Risk Factors Surveillance System (see www.cdc.gov) or other surveys conducted by agencies whose role is to provide knowledge to assist governments in the development of better social-, health-, and work-related policies.

In order to assist agencies that use health surveillance systems, a critical question in the evaluation of outcomes of public mental health interventions is how best to describe mental health of citizens in such a way that allows for inclusion of a broad range of mental health conditions. Recently, it was suggested to use measures of subjective well-being (SWB) to make mental health-related evaluations and inform policy decisions (10).

Currently, there are several projects conducted by national statistical offices and agencies around the world designed to enhance the use of subjective measures of well-being, including also measures of satisfaction with life, as basic indicators. For example, the European agency Eurofound (https://www.eurofound.europa.eu/data/european-quality-of-life-survey) and the UK office of National Statistics (11) used satisfaction with life evaluation as a subjective indicator at the national level on a regular basis, in the framework of surveys that include consultation with general population samples. In 2013, European member-states agreed on a single measure, life satisfaction, for monitoring the progress of SWB in the World Health Organization’s health policy framework, “Health 2020” (12).

Life satisfaction is a subjective, cognitive evaluation of an individual’s life as a whole based on the fit between personal goals and achievements (13). It is an indicator of SWB, which is one of the main dimensions of mental health (14). It has been also referred to as a component and a crucial indicator of quality of life (QOL) (15). The most widely used instrument in the field is the Satisfaction With Life Scale (SWLS) developed by Diener et al. (16). Satisfaction with life has gained attention due to its associations with good health outcomes (17), including longevity (18). At opposite, dissatisfaction with life has been related with several poor health outcomes, including long-lasting negative health outcomes. For example, in some studies, life dissatisfaction predicted increased long-term morbidity, work disability, and mortality (19–22). Low levels of average national life satisfaction were also related to a higher prevalence of suicide (23, 24). Psychiatric comorbidities and the duration of a disorder have an inverse relationship with life satisfaction (25). Life dissatisfaction was strongly associated with mood disorders in both patient (20, 26) and general population samples (27). Long-term life dissatisfaction predicted the onset of MDD in a 7-year longitudinal study (28).

Therefore, the use of the life satisfaction indicator appears to be important to determine its sensitivity over time to the implementation of different health and social service provisions or improvements in health policies. At the same time, it could also be particularly useful to identify the vulnerable groups at risk for depression that represents, as said, a major public health concern. Consistently, some authors even suggested using SWB screening, which includes life satisfaction assessment, as an innovative psychopathology prevention strategy, for capturing individuals at risk for the development of psychopathology, before symptoms onset, based on a low score on SWB (29, 30).

However, the possible use of life satisfaction screening as a prevention strategy to target also depression should be relied on the evidence that SWB is strongly associated with depressive symptoms both among female and male gender and throughout the lifespan.

Concerning the relationships between depression and life satisfaction in the general population, few studies have investigated the influence of gender on their association. Taken together, depressive symptoms appear related to life satisfaction, but the pattern of associations among gender is still debated (31). Females are twice as likely as males to experience depression (32). Thus, it is reasonable to expect that men and women significantly differ in their levels of life satisfaction and in particular that women report lower levels of life satisfaction than men. However, broadly, large-scale nationally representative studies have revealed inconsistent findings on gender differences in life satisfaction, and recent meta-analyses have also resulted in mixed findings (33). Taken together, the conclusions of meta-analyses on this issue, despite contradictory results and a substantial lack of consensus, suggest that at an aggregate level there are no significant gender differences in life satisfaction (33); however, it was recently noted that future studies should be done to disentangle the inconsistencies regarding gender differences in SWB (33).

Among patient population samples, overall, patients who suffer from depression are reported to have lower QOL than the general population (34). It is noteworthy that in this area the available literature mainly concerns QOL, not specifically life satisfaction. While it was suggested that gender differences might be considered an important factor in QOL of depressed patients, there is no consistent conclusion yet (35). Some authors advanced no gender difference in QOL (36), whereas some others reported that female gender played a significant role for better QOL among patients with depression (37) or with bipolar disorder (38). Recently, a study conducted on a large sample of outpatients suffering from residual symptoms of depression found that men had poorer QOL (as measured with the Quality of Life Enjoyment and Satisfaction Questionnaire; Q-LES-Q) and more severe function impairment compared with women (35). This led us to hypothesize that the association of depression with QOL and satisfaction might be different in males and females.

Twin studies may contribute to understand the gender specificities underlying the association between life satisfaction and depression. Recently, there was a growing interest in evaluating the role of shared genetic and environmental influences in the relationship between depressive symptoms and satisfaction with life by gender, even though limited to some studies conducted on adolescent (29, 39) and young adult individuals (40, 41). While these studies consistently showed a substantial role of genetic factors in explaining the correlation between satisfaction with life and depressive symptoms, as regard gender-specific effects they also resulted in mixed findings. Some studies (39, 41) found no evidence for gender differences in the magnitude of the genetic and environmental effects, while others indicated gender-specific effects (29, 40). Therefore, it remains unclear if gender really matters for individual differences in the overlap between life satisfaction and depression.

In the light of the ascertained relationships of life satisfaction with depression and other indicators of poor mental health (28) and of the uncertainty about gender differences in this relationship, we designed this study to investigate whether satisfaction with life was a useful proximal indicator of depressive condition among both male and female individuals. Specifically, the objective of the present study was to investigate whether satisfaction with life was related to depressive symptoms depending on gender, in a sample consisting of women and men, some of whom screened positive for a depressive disorder.

## Materials and Methods

### Study Setting and Subject Characteristics

This study represented a part of the Screening and Enhanced Treatment for DEpression in Primary care (SET-DEP) study (7), whose main objective was to test the feasibility and effectiveness of a program for early detection and treatment of depression in PC. Participants were recruited from 13 urban general internal medicine PC practices, located in central Rome, Italy, from January 2009 to June 2010, and screened for depression. The study inclusion criteria were age 18–65 and absence of psychosis or severe cognitive impairment as clinically determined. Subjects willing to participate were administered a socio-demographic data sheet and the Primary Care Screener for Affective Disorders (PC-SAD) (42). A total of 409 out of 416 subjects who underwent the SET-DEP screening returned a usable PC-SAD with enough answers to be scored.

Afterwards, for other research purposes, a 16-item validated questionnaire on the knowledge and preferences about bio-banking donation (43) and a short questionnaire, which included some questions on self-perceived health and the SWLS (16), were posted to those 409 subjects. Out of the 402 subjects who received the posted scale and questionnaires, 51 responded (13%).

Protocols were approved by the ethical committee of the Italian National Institute of Health, and written informed consent was obtained from all these subjects.

### Measures

The PC-SAD (44) is a short, self-administered questionnaire, which consists of a three-item pre-screener including one Dys question and two MDD questions (which reduce respondent burden by terminating the questionnaire if all are negative), a 26-item MDD section, and an 8-item Dys section. The PC-SAD breaks down each *Diagnostic and Statistical Manual of Mental Disorders, Fourth Edition* (DSM-IV) symptom of MDD into several simple items, and it is scored using an automated system. The scoring algorithm is built in a way that the presence of each symptom can be determined independently from the presence of missing answers to one or more relevant items, provided that at least one item related to the symptom has been answered. The Dys section is scored in a similar way. In the present study, the validated Italian version of the PC-SAD was used (42).

The SWLS in the Italian version by Di Fabio and Gori (45) was used to evaluate life satisfaction. The SWLS (16) is a five-item scale designed to measure the cognitive subjective component of well-being or satisfaction with life (SWL). For each item, participants rated the extent to which they felt generally satisfied with life on a seven-point rating scale (from 1 = strongly disagree to 7 = strongly agree). An example of an item for this scale is “In most ways, my life is close to my ideal.” Scores range from 5 to 35, with higher scores showing greater life satisfaction.

### Statistical Analysis

Descriptive analysis by gender was performed using means with SDs and percentages as appropriate. Gender differences were explored using independent *t*-test for continuous variables (i.e., age and SWLS) and chi-square test for categorical variables (i.e., marital status, education, self-perceived health, and positivity for MDD or Dys as detected by PC-SAD). Interactive effects in SWL between clinical condition and gender were initially explored by looking at gender differences in mean levels of SWLS score in individuals positive and negative at the screening test for MDD or Dys, separately. The effect size of the difference in life satisfaction between males and females positive for MDD or Dys was calculated as measured by Cohen’s *d*. Then, given the small size of this sub-sample, a *post hoc* power analysis was performed.

Because this descriptive approach revealed possible interaction, a multiple linear regression model with SWLS score as the outcome was fitted considering screening status for MDD or Dys, gender, and their interaction as predictors, and further adjusting by age, education level, and perceived health.

Finally, to evaluate whether the interactive effect of depression and gender was present only in depressed males or was also present in females, though to a much smaller degree, the differences in mean levels of SWLS score between participants positive at the screening test for MDD or Dys and participants negative at the screening were tested, both among males and females, separately. Stata software (version 13.1) was used for all analyses except for power analysis for which G*Power 3 software was used (46).

## Results

### Characteristics of Study Participants

Socio-demographic and clinical characteristics of study participants are presented in **Table 1**. The age of participants ranged between 19 and 66 years. There were no significant differences between the female and male groups in age, marital status, education level, and self-perceived health. No significant differences in main socio-demographic variables (age and gender) and clinical characteristics (as percentages of individuals positive for MMD or Dys) were found between participants to the study and those who did not participate (351 individuals out of the 402 who received the satisfaction with life scale).

**Table 1:** Socio-demographic and clinical characteristics of participants.

	Males (*N* = 12)	Females (*N* = 39)	*p* values
**Age** (mean ± SD)	46 ± 12.2	49.6 ± 12.4	0.39
**Marital status** ^§^			
Never married	3 (25.0%)	11 (28.9%)	
Married or living with a partner	9 (75%)	21 (55.3%)	
Separated, divorced, or widowed	0 (0%)	6 (15.8%)	0.28
**Education**			
Primary school	0 (0%)	0 (0%)	
Junior high school	0 (0%)	2 (5.1%)	
Senior high school	6 (50.0%)	21 (53.9%)	
University degree	6 (50.0%)	16 (41.0%)	0.67
**Self-perceived health**			
Bad	1 (8.3%)	5 (12.8%)	
Neither good nor bad	7 (58.3%)	20 (51.3%)	
Good	4 (33.3%)	12 (30.8%)	
Excellent	0 (0%)	2 (5.1%)	0.83
SWL* (mean ± SD)	19.4 ± 9.2	23.7 ± 6.1	0.07^
**Subjects positive for MDD or Dys, *n* (%)**	5 (41.7%)	16 (41.0%)	0.97
SWL* among subjects negative for MDD or Dys (mean ± SD)	^a^26.0 ± 3.4	^b^25.4 ± 4.8	0.77
SWL* among subjects positive for MDD or Dys (mean ± SD)	^a^10.2 ± 5.8	^b^21.2 ± 7.0	0.005

^§^One missing observation.

*SWL, Satisfaction with life.

^This value becomes significant (i.e., 0.04) when adjusting by age in a linear regression model.

^a^p = 0.0001, ^b^p = 0.03 (t test by screening status).

### Relationships Between Positivity for Depression and Satisfaction With Life

Both male and female individuals showed SWL levels in the average score range (the SWLS average score is 20–24), denoting sufficient satisfaction with life. Regarding the PC-SAD scores, men displayed similar depression condition compared with women, with about 42% of subjects positive for MDD or Dys among males (*N* = 5) and 41% among females (*N* = 16) (**Table 1**). Among individuals who screened negative for MDD or Dys (*N* = 30), women (*N* = 23) displayed similar SWLS mean score compared with men (*N* = 7); these scores denoted high satisfaction.

Among individuals who screened positive for MDD or Dys (*N* = 21), women showed greater satisfaction compared with men, whose score denoted dissatisfaction with life. The gender difference in levels of life satisfaction among this sub-sample was highly significant (*p* = 0.005). The effect size of this difference was 1.63. This large effect size *d* of 1.63 gave a study’s power of 84%.

**Figure 1:**
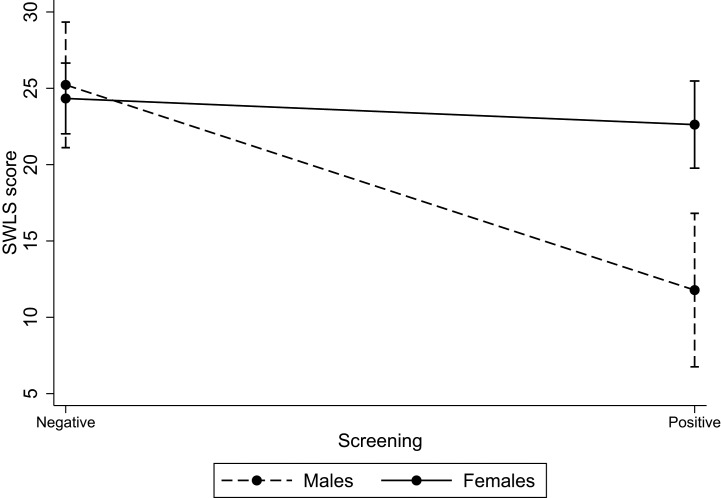
Interaction between positivity for major depressive disorder (MDD) or dysthymic disorder (Dys) and gender on SWLS score. *Note*: The graph is obtained by plotting the predictive margins of the regression model reported in **Table 2**. SWLS, Satisfaction with life scale. Vertical bars indicate 95% CIs.

Accordingly, the regression model for SWLS—adjusted by age, educational level, and self-perceived health—highlighted a main effect of positivity for MDD or Dys on life satisfaction (*p* = 0.000) and a significant interaction between positivity for MDD or Dys and gender (*p* = 0.003). The results of the regression model are reported in **Table 2** and graphically displayed in **Figure 1**.

**Table 2:** Results of the regression model for SWL.

Explanatory variables	Coef.	Std. Err.	*t*	*p* value	95% CI
**Screening** (positive vs. negative)	−13.44	3.30	−4.08	0.000	(−20.09, −6.79)
**Gender** (F vs. M)	−0.89	2.31	−0.38	0.703	(−5.56, 3.78)
Screening*Gender	11.73	3.73	3.14	0.003	(4.20, 19.26)
**Age** (years)	0.01	0.07	0.18	0.857	(−0.14, 0.16)
**Education** (school years)	0.31	0.24	1.30	0.201	(−0.17, 0.79)
**Self-perceived health**					
Neither good nor bad (vs. bad)	−0.77	2.52	−0.31	0.760	(−5.85, 4.31)
Good (vs. bad)	3.95	2.98	1.32	0.192	(−2.07, 9.97)
Excellent (vs. bad)	9.03	4.90	1.84	0.073	(−0.87, 18.93)
**Intercept**	18.59	6.28	2.96	0.005	(5.92, 31.27)

Coef., Estimated beta coefficient; Std. Err., Standard error of the estimate; t, t statistic; 95% CI, 95% confidence interval;

Screening*Gender, Interaction term between positivity for MDD or Dys and gender.

The difference in levels of life satisfaction between men negative at the screening test for MDD or Dys and men positive (in **Table 1**: 26.0 ± 3.4 vs. 10.2 ± 5.8) was strong and highly significant (*p* = 0.0001), whereas the difference in levels of life satisfaction between women negative at the screening test for MDD or Dys and women positive (in **Table 1**: 25.4 ± 4.8 vs. 21.2 ± 7.0) was modest and almost borderline significant (*p* = 0.03). These results together with those of the regression analysis suggested that the relationship between positivity for MDD or Dys and life satisfaction was much stronger in males than in females.

## Discussion

The aim of this study was to further elucidate the association between life satisfaction and depressive symptoms across genders. The analyses that we performed showed a significant difference in life satisfaction between individuals with depressive symptoms and individuals without depressive symptoms and a significant interaction between gender and depressive symptoms, with an effect of depression symptoms on life satisfaction much stronger in male than in female individuals. The lack of a robust association between satisfaction with life and depressive symptoms among female individuals in the present study casts some doubts about the notion that satisfaction with life and depressive symptoms belong to highly associated dimensions, at least among women. The other descriptive analyses on all the individuals regardless of positivity for depression were consistent with the extensive literature reporting no gender differences in life satisfaction (25, 47) and self-perceived health level (48). Moreover, the percentage of individuals who were positive for depression is rather consistent with the percentage found in the former sample of SET-DEP study from which the present study originated (41% and 37%, respectively).

While most previous studies on this topic were performed on young individuals, we examined the relationship between depression and life satisfaction in midlife. The preceding studies have used other screeners or subscales, such as Child Behavior Checklist (CBCL), Youth Self Report (YSR), and Adult Self Report (ASR) *Anxiety and Depressive symptoms* subscales (49). Though the PC-SAD, as well as all previously used screeners, is not a diagnostic instrument, it shows excellent sensitivity and good specificity for the diagnosis of MDD as determined by Structured Clinical Interview for DSM-IV Axis I Disorders (SCID-I), which is regarded as the “gold standard” for making DSM-IV diagnoses (50). Also, both the likelihood ratio positive and likelihood ratio negative were better than the median values reported for other instruments (50). Finally, the sensitivity of PC-SAD was found to be higher than the sensitivity reported for other commonly used screeners (42), such as Patient Health Questionnaire (PHQ) (51) and General Health Questionnaire-12 (GHQ-12) (52).

The findings of the present study are in agreement with recent research conducted on large samples of patients suffering from residual symptoms of MDD (35) and patients suffering from current MDD or bipolar disorder, which indicates that female gender was associated with a better QOL, as measured through Q-LES-Q (38). In our opinion, our findings are also in general agreement with some previous studies, which revealed that female patients reported higher SWB than male patients, although these patients were not affected from MDD but other mental disorders (i.e., schizophrenic and schizoaffective disorders) (53, 54).

In our opinion, the present study corroborates the notion that there are substantial differences between measures of mental health and measures of mental well-being. It was already recognized that SWB, although related, may not coincide with the absence of mental disorders such as depression or anxiety, in that it consists of a global and subjectively weighted evaluation of the individual’s entire life (55). In fact, some previous studies have demonstrated, for example, the limited correlation between positive psychotic symptoms and level of life satisfaction (56). While our finding is in need of further examination, a possible explanation is that women with depression, although concerned about their depressive condition, did not perceive this mental health limitation as related to other aspects of their SWB. Furthermore, it could be that depressed women are more likely than depressed men to seek help and support and have better social networks. Another possible explanation is that depressive symptoms generate higher level of distress in men compared to women, for example, in terms of work, responsibility, or sexual life (35), which in turn might affect their satisfaction with life to a greater extent.

This pattern of results also suggests that life satisfaction is a domain of SWB that is less sensitive to the experiences of depression among women, and dictates further reflection about the practical application and usefulness of satisfaction with life measures for evaluating the outcomes of mental healthcare policy and clinical interventions in female individuals.

As said, the current literature mostly lacks a perspective on the relationship between satisfaction with life and depressive symptoms by gender, except for some recent studies, which include also genetic studies in which phenotypic, genetic, and environmental correlations between satisfaction with life and psychopathology were obtained from multivariate genetic modelling conditional on gender. We consider our findings consistent in particular with those of a twin study (40) showing that genetic and environmental sources of life satisfaction and symptoms of depression were mostly shared, but the magnitude of the effects was different in males and females, with smaller genetic contributions and a more substantial role of shared environmental influences in female compared to male individuals. That is, the extent to which depression and life satisfaction were influenced by the same genes was higher in male than in female individuals. If in male individuals the common genetic component of depression and satisfaction with life is indeed preponderant, it is likely that symptoms of depression tend to correlate with dissatisfaction with life. In female individuals, on the contrary, as a result of various forces, i.e., shared genetic influences and substantial non-shared environmental influences, depressive symptoms and dissatisfaction with life do not necessarily coexist and different conditions can take place, including the presence of depressive symptoms in the absence of a poor satisfaction with life.

Our findings should be viewed in light of some methodological limitations. First, the study included a small sample, which limits the statistical power as well as the generalizability of the results that should be regarded as preliminary and in need of replication with a larger sample. It should be noted that the sample size, though small, was nevertheless sufficient to detect a large difference in life satisfaction between males and females. However, it should be acknowledged that such a sample would likely be insufficient to detect smaller effects (e.g., Cohen’s *d* < 1.00), as well as to test for interactions. Second, the study had a low response rate. It is noteworthy that the request to participate in the present study overlapped with other requests and assessments, which may have strained the potential participants. In fact, in the SET-DEP study, the individuals willing to participate to the study (*N* = 409) were first asked to complete the PC-SAD; then, after 3 months, those who were positive for MDD or Dys and randomized to either the intervention group or the control group in the effectiveness study (*N* = 115) were asked to participate in a telephone follow-up interview in which PC-SAD5 (57) and World Health Organization Quality of Life Brief Version (WHOQOL-Bref) (58) were administered. More or less at the same time of the follow-up interview or just after, the present study was launched with the initial participants of SET-DEP study as target population (*N* = 409). Moreover, it is important to note that the structure of our sample in terms of age, gender, and percentage of individuals positive for MDD or Dys was comparable to that of the reference SET-DEP cohort, and this may provide partial reassurance against severe selection biases.

Third, the study included a low number of men, although this reflected the low number and percentage of men in the former group of SET-DEP study from which the sample of the present study originated (7). On the other hand, the detection of statistically significant gender differences and significant interactive effects between positivity for MDD or Dys and gender despite such a small number of men indicates a large and much more evident effect of male gender, compared to female gender, in the relationship between depressive symptoms and satisfaction with life.

## Conclusions

Clearly, life satisfaction is a key component in the attainment of positive mental health and is a determinant of many life outcomes. Cumulative evidence is required in order to further discover causal pathways through which socio-demographic and environmental factors influence how individuals perceive their satisfaction with life. Specifically, the literature demonstrates the need for further research among adult general population across genders. Additionally, there is a dearth of research that has examined life satisfaction as it pertains to populations with psychopathological conditions.

Also, the implications of the various ways in which knowledge about life satisfaction can aid in the evaluation, implementation, and assessment of programs designed to repair mental health should be stressed. The limited overlap between satisfaction with life and symptoms of depression in the present study only partially justifies the integration of health promotion programs designed to strengthen SWB, as suggested by the World Health Organization, to reduce mental illness (59, 60). Consistently, some literature suggests that interventions targeting well-being may not necessarily have a direct impact on depressive symptoms (39, 55, 61). It is therefore possible that different strategies are needed for preventing mental illness and promoting mental health at the same time, which could be achieved, for example, by assessing both the risk for low SWB and the risk for psychopathology. It should be noted that, to date, depression has been largely ignored as a condition to be monitored to assess the potential impact of any new policy on the mental health of general population. Thus, we hope that these considerations may stimulate further research to better clarify the relationship between satisfaction with life and depressive symptoms across genders.

## Data Availability Statement

The raw data supporting the conclusions of this manuscript will be made available by the authors, after anonymization, without undue reservation, to any qualified researcher.

## Ethics Statement

This study was approved by the ethics committee at the Italian National Institute of Health on June 17, 2008, and written informed consent was obtained from all the participants.

## Author Contributions

AG contributed to the design of the study and the coordination and supervision of data collection and drafted the manuscript. CF contributed to study conception, performed data quality control and statistical analysis, and revised the manuscript for important intellectual content. AG and CF contributed equally to the manuscript. VT contributed to the study conception, coordination, and supervision of data collection and revised the manuscript for important intellectual content. MAS revised the manuscript for important intellectual content. FL revised the manuscript for important intellectual content. CV revised the manuscript for important intellectual content. AP provided inputs for study design, contributed to the supervision of data collection, and revised the manuscript for important intellectual content.

All authors contributed to, read, and approved the submitted version.

## Conflict of Interest Statement

The authors declare that the research was conducted in the absence of any commercial or financial relationships that could be construed as a potential conflict of interest.
